# Efficacy evaluation of low-dose aspirin in IVF/ICSI patients evidence from 13 RCTs: A systematic review and meta-analysis

**DOI:** 10.1097/MD.0000000000007720

**Published:** 2017-09-15

**Authors:** Liping Wang, Xiaman Huang, Xueli Li, Fang Lv, Xiao He, Yu Pan, Li Wang, Xiaomei Zhang

**Affiliations:** a Department of Biobank, Clinical Medical College,Yangzhou University, Northern Jiangsu Province Hospital, Yangzhou; b Department of Obstetrical, the First Affiliated Hospital of Jinan University, Guangzhou, Guangdong; c Reproductive Medicine Center, Department of Obstetrics and Gynecology, Clinical Medical College, Yangzhou University, Northern Jiangsu Province Hospital, Yangzhou, China; d The University of Texas MD Anderson Cancer Center, Department of Anesthesiology & Perioperative Medicine, Houston, TX.

**Keywords:** aspirin, assisted reproductive techniques, clinical pregnancy rate, in vitro fertility, systematic review

## Abstract

**Background:**

We conducted a systematic review and meta-analysis of existing literature to evaluate the different outcomes of low-dose aspirin on patients undergoing in vitro fertilization (IVF)/intracytoplasmic sperm injection (ICSI), including clinical pregnancy rate, implantation rate, live birth rate, miscarriage rate, fertilization rate, number of oocytes retrieved, and so forth.

**Methods:**

Electronic databases including PubMed, MEDLINE, and Embase were searched between 1997 and March 2016 to identity eligible studies. The following comparisons between treatment groups were included: aspirin versus placebo; aspirin versus control group; aspirin versus aspirin + prednisolone + control.

**Results:**

Thirteen randomized controlled trials which included 3104 participants were selected. There were no significant differences in implantation rate (RR = 1.15; 95% CI = 0.78–1.70), live birth rate (RR = 1.06; 95% CI = 0.93–1.21), miscarriage rate (RR = 1.28; 95% CI = 0.93–1.77), fertilization rate (RR = 0.91; 95% CI = 0.75–1.11), and endometrial thickness (WMD = 0.15; 95% CI = −0.38–0.67). But the research showed that aspirin treatment may improve the clinical pregnancy rate (RR = 1.16; 95% CI = 1.04–1.28) compared to placebo or no treatment, and reduce the number of oocytes retrieved (WMD = −0.68; 95% CI = −0.91–0.46).

**Conclusions:**

Our findings suggest that low-dose aspirin may improve the pregnancy rate in IVF/ICSI, with the recommended clinical use dose of 100 mg/day. Considering the limitation of included studies, further well-designed large-scaled RCTs are necessary to clarify whether aspirin may improve assisted reproduction outcomes in IVF/ICSI patients.

## 1 Introduction

Assisted reproductive technology (ART) development, which has had more than 30 years of history, has been utilized all over the world, as millions of “test-tube babies” have been born. ART has become a common way of reliable and effective treatment of infertility, and many infertile couples have benefited from the progress and development of the ART. Although the ART has made a certain progress, the implantation rate and clinical pregnancy rate is still low.^[1]^ How to further improve the clinical pregnancy rate is a constant challenge in reproductive medicine. Researchers test many auxiliary drug interventions for adjuvant therapy, with one of the most clinical effective being aspirin.^[2,3]^

Aspirin also called acetylsalicylic acid and has many clinical applications, including as an antipyretic and analgesic, inhibition, prevent thrombosis and improve microcirculation. It is widely used in clinical fields, including the treatment of cardiovascular diseases, gynecological diseases, and so on. With the development of medical science, more and more experiments have established that aspirin play an important role in infertility and ART. However, there remains uncertainty about, but its effectiveness and the dose-dependent effectiveness. This paper aims to explore the effect of low-dose aspirin in assisted reproductive outcomes through meta-analysis of 13 randomized controlled trials (RCT) articles.

## 2 Materials and methods

### 2.1 Data source and search strategy

This meta-analysis do not involve patients, and thus do not require institutional review board approval. Electronic databases including PubMed, MEDLINE, and Embase were searched between 1997 and March 2016 to identity eligible studies, using the following key words: “aspirin or acetylsalicylic acid,” “IVF or in vitro fertilization,” “RCT or randomized controlled trials,” and “ART or assisted reproductive techniques.” Furthermore, the reference lists of every article were retrieved and reviews were manually searched to identify additional eligible studies.

### 2.2 Eligibility criteria

Studies were included if they met the following criteria: They had to be RCT; the study population included women undergoing IVF or intracytoplasmic sperm injection (ICSI); the clinical outcome of implantation, miscarriage, pregnancy, and live birth was recorded for all participants; low-dose aspirin (<120 mg) was used compared with placebo or no treatment. Review articles, case reports, letters to the editor, and editor comments were excluded.

### 2.3 Data collection

Eligibility evaluation and data abstraction were analyzed independently by 2 investigators (Liping Wang, Xiaman Huang) according to the meta-analysis observational studies in PRISMA guidelines, and the discrepancies were adjudicated by consensus. For each study, the following data were extracted: first author; year of publication; country; included patients (aspirin/placebo); blinding; treatment and dose; inclusion criteria; type of analysis in original article; outcome measures (clinical pregnancy rate; implantation rate; live birth rate; miscarriage rate; fertilization rate; endometrial thickness; oocytes retrieved).

### 2.4 Statistical analysis

All results were merged for meta-analysis with the Review Manager 5.2 (version 5.2, The Nordic Cochrane Centre, The Cochrane Collaboration, 2016). The number of women who were randomly assigned was regarded as the total number of participants in each study. Using the Mantel–Haenszel random-effects model and Fixed model dichotomous outcomes were summarized by calculating the relative risk (RR) and 95% confidence intervals (95% CIs) and Weighted mean difference (WMD). Heterogeneity between studies was assessed by the Chi-squared test and *I*^2^ with *P* < .10 indicating significant heterogeneity. *I*^2^ results interpretation: *I*^2^ < 25%: low heterogeneity; *I*^2^ = 25% to 50%: moderate heterogeneity; *I*^2^ > 50%: a high degree of heterogeneity.

## 3 Results

**Figure 1:**
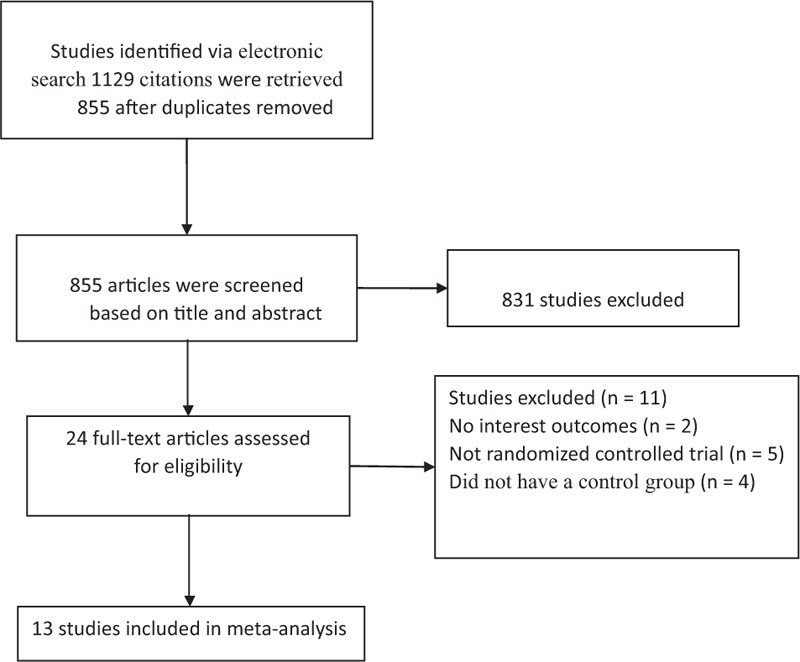
Flowchart of study selection.

The electronic search retrieved a total of 1129 citations. (Aspirin or acetylsalicylic acid) AND (IVF or in vitro fertilization) and (RCT or randomized controlled trials) AND (ART or assisted reproductive techniques.) Of these, 855 articles were excluded on the basis of title and abstract; 24 articles were assessed fully for eligibility. A total of 11 articles were excluded for the following reasons: the effect of aspirin after treatment had not been evaluated in terms of ART outcomes (n = 2); the study was not randomized controlled trial (n = 5); no control group (n = 4). Therefore, 13 studies were finally included in this meta-analysis (Fig. 1). The characteristics of the included studies are listed in Table 1.^[2,4–15]^

**Table 1:**
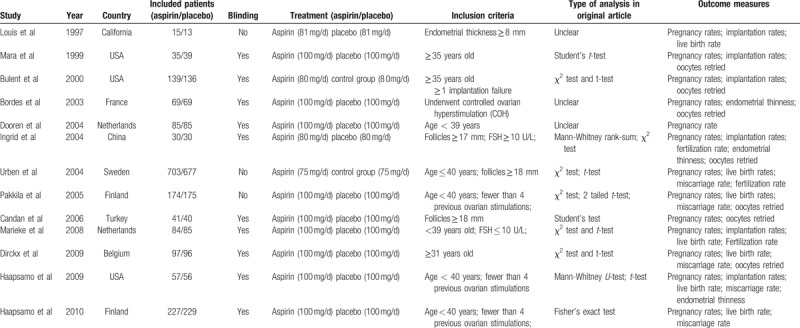
The characteristics of included studies.

**Figure 2:**
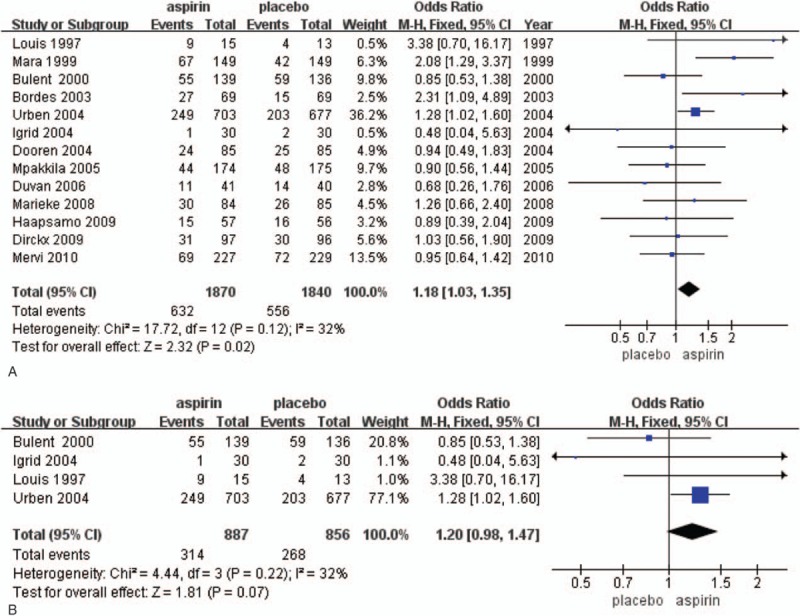
The Forest plots for (A) clinical pregnancy rate, (B) the improved clinical pregnancy rate.

The process of randomization was appropriate in 13 studies. Allocation concealment was carried out in 13 studies, which may give rise to selection bias. The researchers and participants were blinded to the intervention in 13 studies. For outcome assessment, there were drop outs in 2 studies, and the reason was reported. Results of quality assessment of the studies are shown in Fig. 2.

### 3.1 Clinical pregnancy rate

All 13 studies (3104 participants) that reported clinical pregnancy as an outcome were included (Fig. 2A). Clinical pregnancy occurred in 531 of 1565 (33.9%) women randomized to low-dose (75–100 mg/d) aspirin and 449 of 1539 (29.1%) women randomized to placebo or no treatment. The pooled analysis using the random-effects model demonstrated that low-dose aspirin use might improve the clinical pregnancy rate compared with placebo or no treatment group (RR = 1.16; 95% CI = 1.04–1.28; *P* = .005). There was significant heterogeneity between studies (*I*^2^ = 19%).

The 4 papers studying (1743 participants) a lower dose of aspirin (75–80 mg/d) found that it may also improve the clinical pregnancy rate compared with placebo or no treatment group using the random-effects model analysis (Fig. 2B). But there was no significant heterogeneity between studies (RR = 1.13; 95% CI = 0.99–1.29; *P* = .07) (*I*^2^ = 19%).

### 3.2 Number of oocytes retrieved

**Figure 3:**
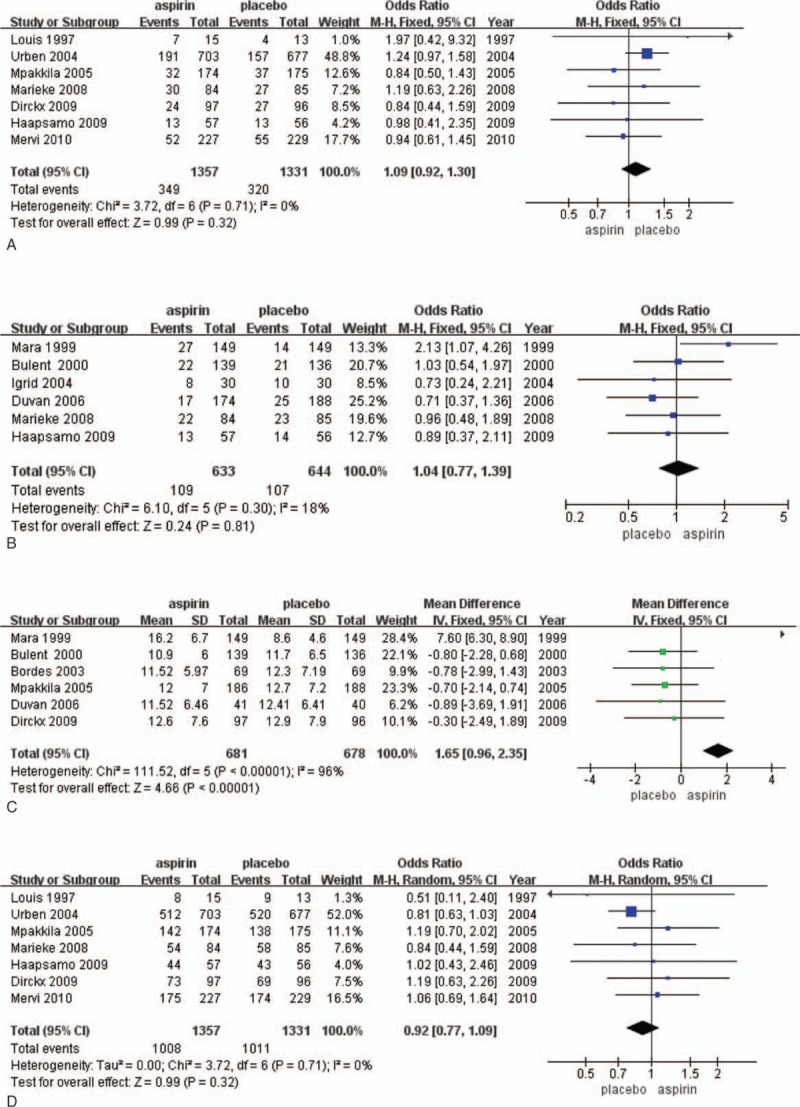
The Forest plots for (A) number of oocytes retrieved, (B) implantation rate, (C) live birth rate, (D) miscarriage rate.

There were 5 studies (1061 patients) which reported the number of oocytes retrieved. The random-effect model was used for the meta-analysis. The results showed that the number of oocytes retrieved of the aspirin was significantly higher than that of the placebo or no treatment groups (MD = −0.70; 95% CI = −1.52–0.12; *P* = 1.00), while no significant heterogeneity was found (*I*^2^ = 0%) (Fig. 3A).

### 3.3 Implantation rate

Seven studies reported implantation rate outcomes in 1240 patients. Implantation occurred in 102 of 612 (16.7%) women randomized to low-dose aspirin and in 90 of 628 (14.3%) women randomized to placebo or no treatment. Thus, the random-effect model was used for the meta-analysis. The results showed there was no significant difference in the implantation rate between the aspirin and placebo groups (RR = 1.15; 95% CI = 0.78–1.70; *P* = .07). In terms of single study data, only 2 trials reported a significant increase in implantation rate (Fig. 3B).

### 3.4 Live birth rate

**Figure 4:**
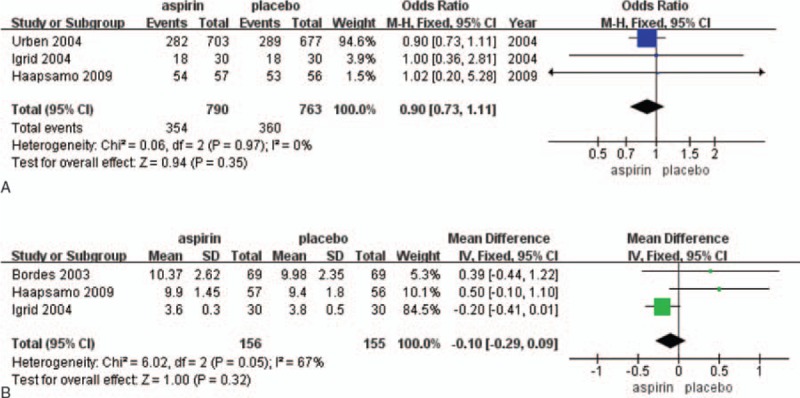
The Forest plots for (A) fertilization rate, (B) endometrial thickness.

There were 7 studies (1372 aspirin patients vs 1347 placebo patients) which reported live birth rate. There was no heterogeneity among the studies (*I*^2^ = 0%) (Fig. 4B). The results showed that there was no significant difference in live birth rate between the aspirin and placebo groups (RR = 1.08; 95% CI = 0.93–1.21; *P* = .74). In terms of single study data, only 2 trials reported a significant increase in birth rate^[2,9]^ (Fig. 3C).

### 3.5 Miscarriage rate

There were 5 studies (1850 participants) which reported on miscarriage rates (Fig. 3D). Miscarriage occurred in 78 of 936 (8.3%) women randomized to low-dose aspirin and in 60 of 914 (6.6%) women randomized to placebo or no treatment. No significant change in the rate of miscarriage was observed between aspirin and placebo or treatment groups. Considering the result from the pooled data analysis, as well as any single study (RR = 1.28; 95% CI = 0.93–1.77; *P* = .53). No significant heterogeneity was detected between studies (*I*^2^ = 0%).

### 3.6 Fertilization rate

The fertilization rate was reported in 3 studies (1609 participants). Fertilization occurred in 353 of 817 (43.2%) women randomized to low-dose aspirin and in 361 of 792 (45.6%) women randomized to placebo or no treatment. Pooling the results of 3 studies showed no significant difference between patients treated with the aspirin compared with the placebo (RR = 0.91; 95% CI = 0.75–1.11; *P* = .95). There was no heterogeneity among the studies (*I*^2^ = 0%) (Fig. 4A).

### 3.7 Endometrial thickness

There were 3 studies (311 participants) that reported on endometrial thickness (Fig. 4A). No significant change in the rate of endometrial thickness was observed between aspirin and placebo or treatment groups. This was true across the result from the pooled data analysis, as well as any single study (RR = 0.15; 95% CI = −0.38–0.67; *P* = .05) (Fig. 4B).

## 4 Discussions

The data of this systematic review and meta-analysis show that low-dose aspirin may improve clinical pregnancy rate in IVF/ICSI. As infertility treatment success rate remains low, the years tried various methods have been used to improve (IVF/ICSI) outcomes.^[16]^ This research focus on aspirin effects on clinical pregnancy outcomes.

Rubinstei et al have shown that aspirin can effectively inhibit platelet aggregation. The mechanism is through selective acetylation of COX a serine hydroxyl, irreversible inhibition of the cyclooxygenase (COX) enzyme, reducing activity of thromboxane A2 (TXA2) and prostaglandin synthesis (PGs), inflammatory reaction,^[4]^ thus inhibiting platelet activity, and preventing the formation of blood clots, as well as reducing resistance in the blood vessels and increasing tissue perfusion. This systemic and local environment affect the ovarian and endometrial, making it unfavorable to the embryo implantation.^[17]^ As well as this, low-dose aspirin can treat fetal retardation^[18]^ of severe pregnancy, high character,^[19]^ recurrent abortion^[20]^ and uterine ovarian perfusion.^[21]^

Ng et al^[22]^ carried out clinical randomized controlled trials which found that in patients receiving low-dose aspirin therapy IVF cycle and ovary decreasing PI, increased blood flow velocity, human chorionic gonadotropin (hCG) levels, E2 levels, and the number of eggs compared with the control group. Hsieh et al^[23]^ with 3-dimensional ultrasonic testing found that patients with endometrial blood flow in the super ovulation cycle is significantly lower than the natural cycle. Repeatedly failed IVF patients are often due to high endometrial artery blood flow resistance and low blood flow. Aspirin can improve human endometrial thickness, shape, reduce PI, resistance index (RI), increase the amount of blood flow perfusion and increase the rate of lining of 3 wire.^[23]^

In 1991, Testart and Gauthier^[24]^ first use animal experiments to show that aspirin may increase clinical pregnancy rates by use increasing uterine artery blood flow. Therefore, based on animal experiments, we can assume that aspirin was a positive role in patients with low implantation and pregnancy rates. In our study pregnancy occurred in 531 of 1565 (33.9%) women randomized to low-dose aspirin and in 449 of 1539 (29.1%) women randomized to placebo. The pooled analysis using the random-effects model demonstrated that aspirin may improved the clinical pregnancy rate compared with placebo or no treatment groups (RR = 1.16; 95% CI = 1.04–1.28; *P* = .25). It showed that low-dose aspirin also can improve the clinical pregnancy rate of IVF patients, but we have not found the implantation rate to have obvious change.

Academics have found that small doses of aspirin is more effective, as it gives a better ratio of TXA2/PGI2, which results is a greater reduction vascular tone and improve tissue perfusion.^[25]^ Literature shows that 100 mg/day can reach the best biological effect without affecting the blood coagulation time.^[26]^ So the authors of this study choose this as the ideal dose for treatment.

Another study was suggested low doses of aspirin can significantly increase the thickness of the endometrium in patients with clinical pregnancy.^[2]^ Therefore, further studies could be done exploring if increasing endometrial thickness (through increased blood supply promoting endometrial growth) improves endometrial receptivity and the clinical pregnancy rate.

For the first time in 2011, aspirin was studied in nonselective IVF/ICSI follicles cycle with different duration of endometrial thickness and clinical pregnancy outcome. Results showed that aspirin group compared with the control group had significant differences (*P* < .05), and the authors first suggested that it may be through the process of controlling super ovulation (COH). They suggested that aspirin effect time should be greater than 25.65 ± 2.06 days, to increase the transplant the endometrial thickness, and improve endometrial reception of embryos.^[27]^

On March 2016, Liu Juan et al included 50 female patients with infertility due to ovulatory dysfunction and divided them into the study group (aspirin 100 mg/d+ clomiphene) and control group (clomiphene treatment) with 25 patients in each. The results showed that low-dose aspirin therapy helps increase blood supply to the uterus, effectively promoting the development of endometrial thickness, and helping improve the clinical pregnancy rate.^[28]^

Before we start writing this manuscript, we have 19 articles included, now we have 13 RCT articles after eliminate 6 of them not RCT articles in our manuscript. After carefully reading our study, we had been modified the first line of Materials and Methods following with the PRISMA guidelines. In our manuscript all the pregnancy rate means clinical pregnancy rate, it is 4 weeks after transfer, the fetus heartbeat can be monitor by ultrasound. We have already modified all this parameter from pregnancy rate to clinical pregnancy rate. In our manuscript, implantation rates means the number of embryo implantation/the total number of embryos, clinical pregnancy rates means the pregnancy cycles/the number of transplantation cycle. After embryo implantation, it usually takes 14 days to check the heartbeat of the fetus. Aspirin may play a very important role in the maternal adjustment. There was no change in implantation rate, but significantly improve the clinical pregnancy rate. Further studies are required to focus on this aspect to verify that aspirin influence more to the maternal environment than the embryo quality.

In summary, low-dose aspirin is an effective treatment for IVF patients because it can improve the clinical pregnancy rate and number of oocytes retrieved. Further larger RCTs with adequate sample sizes and methodologically rigorous trials, including in various kinds of subgroups (e.g., according to age, endometrial thickness and other outcomes, or in freezing thawing cycle) are required to determine whether there is a difference in effectiveness between low-dose aspirin and placebo.
